# A proteomic evaluation of urinary changes associated with cardiopulmonary bypass

**DOI:** 10.1186/s12014-016-9118-9

**Published:** 2016-08-15

**Authors:** Ravi C. Dwivedi, Mario Navarrete, Nora Choi, Victor Spicer, Claudio Rigatto, Rakesh C. Arora, Oleg Krokhin, Julie Ho, John A. Wilkins

**Affiliations:** 1 Manitoba Centre for Proteomics and Systems Biology, University of Manitoba and Health Sciences Centre, Room 799, John Buhler Research Center, 715 Mc Dermot Avenue, Winnipeg, MB R3E 3P4 Canada; 2 Department of Internal Medicine, Section of Biomedical Proteomics, University of Manitoba, Winnipeg, MB Canada; 3 Department of Internal Medicine, Section of Nephrology, University of Manitoba, Winnipeg, MB Canada; 4 Department of Surgery, University of Manitoba, Winnipeg, MB Canada; 5 Cardiac Sciences Program, St. Boniface Hospital Research Centre, Winnipeg, Canada; 6 Department of Immunology, University of Manitoba, Winnipeg, MB Canada

**Keywords:** Data dependent acquisition (DDA), Information dependent data acquisition (IDA), Data independent acquisition (DIA), Molecular weight cut off (MWCO) filters, Label free quantitation, SWATH, Proteomics, Cardiac surgery, Urine, Renal

## Abstract

**Background:**

The urinary proteome of patients undergoing cardiopulmonary bypass (CPB) may provide important insights into systemic and renal changes associated with the procedure. Such information may ultimately provide a basis to differentiate changes or properties associated with the development of acute kidney injury. While mass spectrometry (MS) analysis offers the potential for in-depth compositional analysis it is often limited in coverage and relative quantitation capacity. The aim of this study was to develop a process flow for the preparation and comparison of the intraoperative urinary proteome.

**Methods:**

Urines were collected from patients at the start of CPB and 1-h into CPB. Pooled samples (n = 5) from each time point were processed using a modified Filter Assisted Sample Preparation protocol. The resulting peptides were analyzed by 2D-LC–MS/MS and by 1D-LC–MS/MS SWATH (Sequential Window acquisition of All Theoretical fragment ion spectra).

**Results:**

The 2D-LC–MS/MS analysis identified 1324 proteins in the two pools, of which 744 were quantifiable. The SWATH approach provided quantitation for 730 proteins, 552 of which overlapped with the common population from the 2D-IDA results. Intensity correlation filtering between the two methods gave 475 proteins for biological interpretation. Proteins displaying greater than threefold changes (>log_2_ 1.59) at 1-hour CPB relative to the initiation of CPB (26 down-regulated and 22 up-regulated) were selected for further analysis. Up-regulated proteins were enriched in GO terms related to humoral immune response, predominantly innate immunity (C4b, lactotransferrin, protein S100-A8, cathelicidin, myeloperoxidase) and extracellular matrix reorganization (e.g. MMP-9).

**Conclusions:**

This study describes a scheme for processing urine from patients undergoing CPB for mass spectrometry-based analysis. The introduction of SWATH into the workflow offers a sample and instrument sparing approach to obtaining consistent in-depth sample analysis. The design of the methodology is such that it can be readily applied to large numbers of clinical samples with the potential for automation. The results also suggest that activation of the innate immune responses occur during cardiac bypass surgery.

**Electronic supplementary material:**

The online version of this article (doi:10.1186/s12014-016-9118-9) contains supplementary material, which is available to authorized users.

## Background

Urine proteomic analysis is a powerful tool that may provide insights into protein changes that are predictive of disease development and to define underlying pathophysiologic processes. Urine provides a non-invasive, rich source of proteins that are potentially informative of systemic and renal processes. Indeed while there are unique aspects to the urine proteome, it has been shown to have approximately 61 % overlap with both the kidney and plasma proteomes [1]. These considerations have been the rationale for a number of proteomic studies of urinary proteins [2–5]. However, urine proteomic analysis is complicated by protein concentration variability, the presence of interfering salts, metabolites and other substances [6]. These considerations often require significant sample processing which can impact the reproducibility of quantitative analysis of urine [7].

Different urine proteomics approaches have been applied to clinical scenarios in the intensive care unit [5, 8], renal transplants [9, 10], and pre/post-operative cardiac surgery [2]. However, proteomic evaluation of intraoperative urines is particularly challenging because of the presence of unique inhibitory materials that interfere with chromatography. Only targeted SELDI TOF–MS analysis has been successfully applied to intra-operative cardiac surgery urine samples [3, 11, 12], however this approach offers extremely limited proteome coverage and does not provide specific protein identification.

Shotgun proteomics on complex biological samples using in-depth 2D-LC–MS/MS analysis provides maximum proteome coverage and protein identification. However, the stochastic nature of DDA/IDA peptide selection for analysis often results in variable identification coverage between analyses [1, 13, 14]. Sequential Window acquisition of All Theoretical fragment ion spectra Mass-Spectrometry (SWATH-MS) has been proposed as an alternative approach [15]. SWATH-MS offers accurate and reproducible protein quantification from complex samples using data-independent acquisition. A significant advantage of SWATH analysis is that it reports the relative abundance of selected proteins in different samples. SWATH-MS also provides a permanent digital file representing the continuous MS-measurable proteome of a sample that can subsequently be re-analyzed as new approaches and insights become available [16].

This study describes a workflow for the processing and analysis of urines from adult patients undergoing cardiac bypass surgery. A comparison of DDA/IDA and SWATH-based analysis is provided suggesting that the latter may represent a useful adjunct for the comparison of clinical samples.

## Methods

### Patients and samples

The study protocol was approved by the Health Research Ethics Board, University of Manitoba and all patients provided informed consent. Patients with stable renal function (n = 5) were selected for analysis from a larger prospective observational cohort of adult cardiac surgery patients using the following criteria: baseline estimated glomerular filtration rate (eGFR) ≥60 mL/min and post-operative serum creatinine rise <10 % from baseline. Serial urine samples were collected at the initiation of CPB and after 1-h on CPB. Urines were spun at 870 g for 6 min, and the supernatants stored at −80 °C for further analysis. The baseline clinical characteristics of the patient cohort are described in Table 1.

**Table 1:** Baseline patient characteristics

Variable	Patients (n = 5)
Age (years)	75 (73–77)
Male	3 (60 %)
eGFR (mL/min/1.72 m^2^)	72.5 (69.2–76.5)
Baseline creatinine (mg/dL)	94 (69–97)
THAKAR Score	2 (1–2)
Diabetes mellitus	1 (20 %)
Chronic obstructive pulmonary disease	1 (20 %)
Hospitalized congestive heart failure	1 (20 %)
Previous myocardial infarction	2 (40 %)
Previous CABG	0 (0 %)
Peripheral arterial disease	1 (20 %)
Amputation or peripheral arterial disease bypass	0 (0 %)
Previous cerebrovascular accident	0 (0 %)
Previous transient ischemic attack	1 (20 %)
Type of surgery (isolated CABG)	3 (60 %)
Pump time (min)	68 (64–95)
Cross-clamp time (min)	54 (36–78)
Operating room duration (min)	254 (250–305)
European system for cardiac operation risk evaluation EuroSCORE II	1.5 % (1.0–2.4 %)
Intraoperative urine output (mL)	1180 (800–1350)

Values expressed as median (interquartile range) or N (percent). Continuous variables compared using Mann–Whitney test, categorical variables compared using Chi-square or Fisher’s Exact Test

### Sample preparation for mass-spectrometry analysis

Total urine protein was quantified using a total Protein Kit, Micro Pyrogallol Red Method (Sigma, USA). A pool of urines from 5 patients (40 µg protein/patient) was prepared for each time point. The pooled samples were processed using a modified FASP protocol [17]. Pooled urines were treated for 5 min with 0.1 M DTT at 100 °C. The cooled samples were brought to 8 M urea, 100 mM Tris pH 8.0 and diluted with an equal volume of the same solution. The samples were transferred to the indicated Amicon MWCO ultra-15 centrifugal filter (Millipore) (i.e. 3, 10 or 30 kDa cut-off) and centrifuged at 4000×*g* for 15 min. The retentate was then diluted to 15 ml with 8 M urea, 100 mM Tris pH 8.0 and centrifuged for 30–50 min at 4000×*g* to remove excess DTT. This was repeated once after which 2 ml of 50 mM iodoacetamide was added to the sample and incubated in the dark for 15 min. The sample was centrifuged for 15 min at 4000×*g* and 2 ml of 20 mM DTT was added to react with residual iodoacetamide. The sample was centrifuged for 15 min at 4000×*g*, after which the retentate was brought to 500 μl with 50 mM ammonium bicarbonate. Trypsin was added at 1:50 ratio based on the starting protein amount and the sample was incubated overnight at 28 °C with shaking. The digest was brought to 500 mM NaCl and gently mixed. The samples were centrifuged for 15 min at 4000×*g*. The MWCO filter was washed with 200 μl of water by centrifugation for 15 min at 4000×*g*. The peptides were eluted from the membrane by centrifugation (3000×*g*, 15 min) with 500 μl of 15 % acetonitrile, 0.1 % trifluoroactic acid in water. The eluted peptides were dried using a speedvac to remove the organic solvents used for elution from the MWCO membranes. The sample was dissolved in 500 μl of 0.5 % trifluoroacetic acid and desalted with a C18-SD extraction disc cartridge (Sigma, USA). The eluted peptides were dried and reconstituted in 40 μl of 0.1 % formic acid in water. An estimation of peptide quantity was made using a Nano-drop spectrophotometer (Thermo Scientific Nanodrop 2000) at 205 nm absorbance where 1.0 absorbance unit corresponded to a peptide concentration of ~1 μg/μl.

## 2D-HPLC–MS/MS data acquisition and peptide identification

The processed trypsin digested peptides were separated using two-dimensional liquid chromatographic method [18]. C-18 cleaned peptides obtained after modified FASP procedure were gradient fractionated on a C18 X-Terra column, (1 × 100 mm, 3 µm, 100 Å, Waters, Milford, MA). Both eluents A (water) and B (90 % acetonitrile) contained 20 mM ammonium formate buffer pH 10.0. A total of 30 fractions were collected using a gradient of 1–44 % of solvent B in 30 min, at a flow rate of 300 µl/min. The resulting fractions were concatenated to eight fractions, dried and dissolved in 50 µl of eluent A (0.1 % Formic Acid). One microgram from each concatenated fraction was injected into a splitless nano-flow Tempo LC system (Eksigent, Dublin, CA) via a PepMap100 trap column (0.3 × 5 mm, 5 µm, 100 Å, Dionex, Sunnyvale, CA) and a 100 μm × 150 mm analytical column packed with 3 μm Luna C18(2) (Phenomenex, Torrance, CA). Both eluents A (2 % acetonitrile in water) and B (98 % acetonitrile) contained 0.1 % formic acid as ion-pairing modifier. A 0.44 % acetonitrile per minute linear gradient (0–35 % B in 80 min, 500 nl/min) was used for peptide elution, followed by 5 min wash with 80 % B.

Samples were analyzed on a 5600 TripleTOF mass spectrometer (SCIEX, Canada) in standard MS/MS data dependent acquisition (DDA) mode also referred as information dependent acquisition (IDA). Each DDA/IDA cycle included a 250 ms MS scan (400–1600 m/z) and up to 20 MS/MS (100 ms each, 100–1600 m/z) for ions state of +2 to +5 and an intensity of minimum 300 cps. Selected ions and their isotopes were dynamically excluded from further fragmentation for 12 s.

For each pooled sample the resulting eight instrument-level WIFF files were converted to MGF format using the conversion tool bundled with Analyst. These were then sequentially concatenated into a single MGF file for each sample pool. Peptide identification and quantitation was done using X!tandem (cyclone 2012.10.01.1) [19]. Peptides were identified against a human database of the June 2015 release of SwissProt with the following search settings: constant modification C +57.021 (cysteine protection); variable modifications: M, W +15.995 (oxidation) or +31.989 (double oxidation); S, T, Y +79.966 (phosphorylation), N, Q +0.984 (deamidation); parent mass error: ±20 PPM, fragment mass error: 0.1 Da. Peptide identification Log_10_ expectation values were computed using a survival function approach [20, 21].

### SWATH-MS acquisition

SWATH-MS, also referred to as data independent acquisition (DIA), runs were conducted using a 5600 TripleTOF mass-spectrometer, which operated in a looped product ion mode. This mode helps instrument to allow quadrupole resolution of 25 amu per mass selection. Precursor selection windows had an overlap of 1 Da with their adjacent selection window to ensure complete isotope coverage between SWATH blocks. Each cycle of SWATH analysis consisted of a 100 ms MS scan and a 100 ms MS/MS scan in 25 m/z blocks in 400–1250 m/z range, and a total of 34 SWATH blocks were collected for each scan. An accumulation time of 100 ms was used for each fragment ion scan and for the survey scans acquired at the beginning of each cycle, resulting in a total cycle time of 3.5 s. The collision energy for each window was determined on the basis of the collision energy for a 2+ ion centered in the respective window (equation: 0.0625 × m/z −3.5) with a collision energy spread of 15 eV. The mass spectrometer was always operated in high sensitivity mode. Peptides were separated using the same LC system configuration as for the second dimension DDA/IDA acquisition, using a 0.30 % acetonitrile per minute linear gradient (0–33 % B in 110 min, 500 nl/min), followed by a 5 min wash with 80 % B.

### SWATH library and analysis

For SWATH library construction all non-modified peptides across a large dataset 2D DDA/IDA runs were concatenated into a non-redundant collection using methods described earlier [22]. We opted to include all peptides from a broader study of eight urine pool samples, regardless of their expectation values or number of peptides per protein detected in each run, to yield maximum SWATH depth. The peptide entries from each DDA/IDA run were retention-aligned against a system average value using the retention times extracted for the custom peptides spiked into each sample.

To enhance our potential SWATH extraction coverage, we developed a method to supplement this library with transitions from the comprehensive human SWATH library [23]. These “10K-library” transitions represented peptide fragment ion patterns detected in SWATH runs, which differ somewhat from their DDA/IDA counterparts due to differences in instrument fragmentation settings across the acquisition methods. This library also provided transition versions for multiple potential charge states of a given peptide, a feature that varies between 1D and 2D runs as a product of finite instrument acquisition time per MS/MS cycle.

This combined library was retention aligned against average retention values acquired from prior DDA/IDA runs under identical chromatographic settings. This library drove the SWATH analysis component of Peakview (1.2.0.4; AB Sciex 2012). The SWATH parameters were a 6 min extraction window (±3 min), a mass accuracy 50 PPM, a search depth of 100 peptides per protein, 6 transitions per peptide, and a 1 % false discovery rate. Extraction was performed on all eight SWATH runs of our larger study simultaneously to permit optimal “gap filling”, yielding a modest transition detection rate of ~11 % (21,205 of 193,112 targeted transitions) spanning 6050 peptides.

The utility of the custom-peptide [24] based retention alignments is illustrated in (Additional file 1: Table S1). The differences between the library target retention times and their actual extracted retention times in both of our SWATH runs are well within our SWATH retention extraction time window with no apparent systematic skewing of the retention differences, indicating good control over the chromatography across the SWATH runs and between the methods.

## Results and discussion

### Optimization of urine sample preparation for mass spectrometry

The aim of the present study was to develop methods for the reliable comparison of the urinary proteome during CPB. Preliminary studies indicated that urine samples collected during cardiac surgery contained substance(s) that interfered with processing and chromatographic separations of the peptides derived from these samples. However, samples collected post operatively from the same patient group did not appear to contain the chromatographic-interfering substances. Although unproven it was felt that the most likely cause of these interfering effects were due to lipophilic substances possibly propofol, an anaesthetic used during the surgery.

**Fig. 1:**
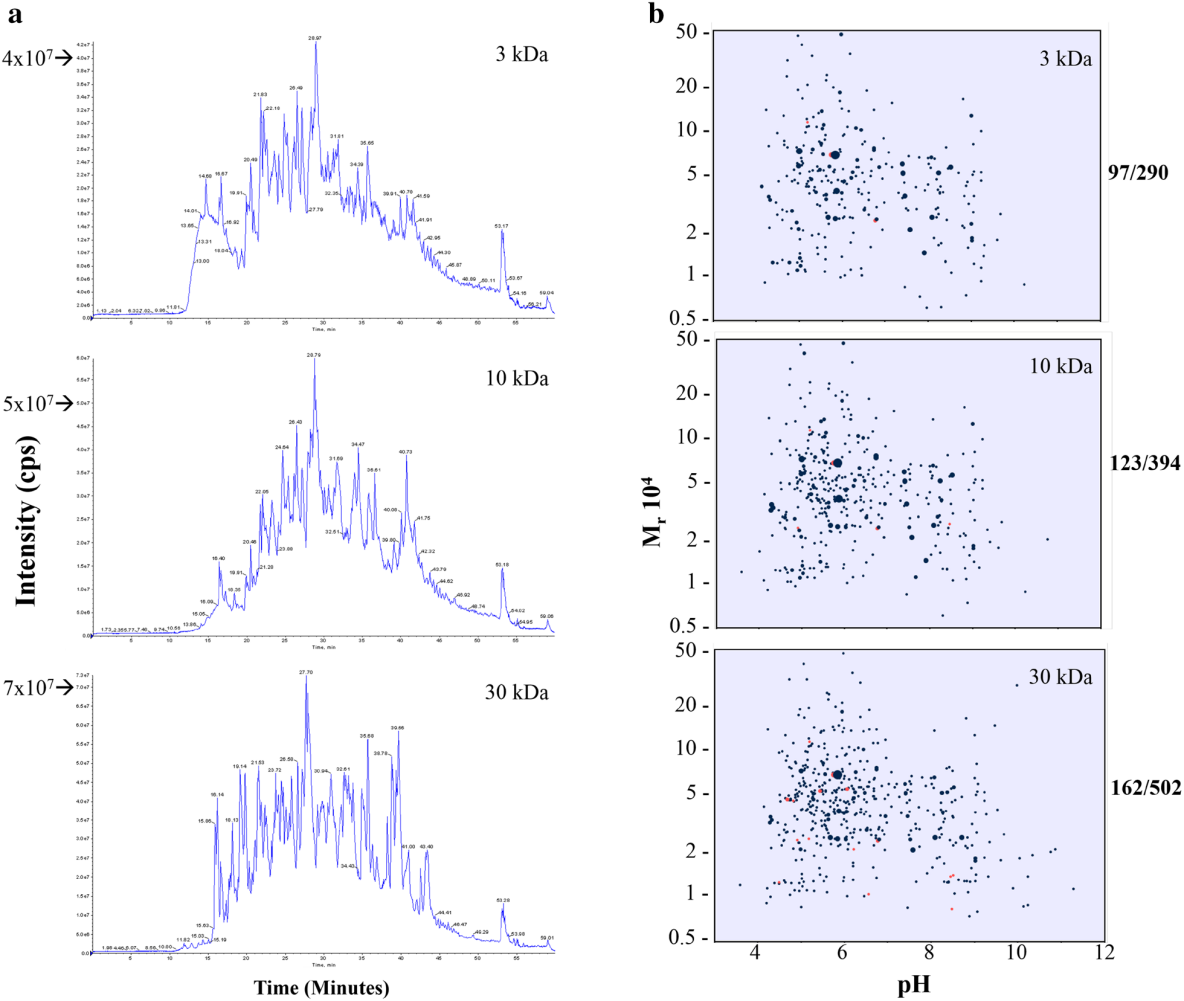
**a** The chromatographic properties of CPB urine samples processed using 3, 10 or 30 kDa MWCO filters in the modified-FASP method. The total ion chromatogram of each sample is given. Note the peak spreading and altered retention times in the 3 and 10 kDa MWCO membrane processed samples. **b** The predicted molecular weight and pI distribution of proteins identified from the same urine sample FASP processed with 3, 10 or 30 kDa molecular weight cut-off membranes. The values beside *each panel* indicate the number of proteins with molecular weights less than 30 kDa and the total number of proteins identified in sample

After examining several approaches we established that a modified version of the filter-aided sample preparation (FASP) procedure [17] provided a suitable approach. This procedure involves sample processing with subsequent concentration of the sample using a molecular weight cut off filter. The concentrated sample is then processed in situ and the resulting peptides are eluted for analysis. This approach allows for sample concentration and clean-up in a single reaction vessel, limiting protein loss and minimizing technical variability. The SDS and urea used in this procedure also offered a means of removing hydrophobic interfering substances which were present in the samples collected from patients during surgery. Preliminary experiments determined that samples prepared with either 3 or 10 kDa cut off filters proposed in the original FASP method [17] still contained compounds that altered the chromatographic properties of the recovered peptides (Fig. 1a). The retention times of peptides isolated under these conditions were reduced and there was considerable peak spreading which resulted in sub-optimal separations. Increasing the pore size of the filters to 30 kDa molecular weight cut off membrane eliminated this effect suggesting that the interfering materials were removed under these conditions (Fig. 1a). However, there was concern that the increased pore size could also result in the selective loss of lower molecular weight species in the urine samples. A comparative analysis of the 30 kDa membrane processed samples with those produced with 3 or 10 kDa membranes indicated that the 30 kDa sample provided a greater number of proteins in the 1–30 kDa range and overall more protein identifications than the other samples (Fig. 1b). While these results could not exclude the possibility of some protein loss using the 30 kDa molecular weight cut off membranes, it was apparent that the recovery and range of proteins was better with these membranes than with either the 3 or 10 kDa membranes. This increased recovery was demonstrated by the higher numbers of protein identifications and the numbers of proteins with molecular weights less than 30 kDa in samples processed using the 30 kDa MWCO filters (502 and 162, respectively) as compared with the 3 kDa (290 and 97) or 10 kDa (394 and 123) MWCO processed samples. The recent results of Berger et al. suggest that perhaps even larger pore PVDF based membranes may be used for sample preparation because of the efficiency of protein capture [25].

### Differential protein analysis

Digests of the samples were analyzed by 2D-LC–MS and peptides were quantified by summing the intensities of all fragments in their associated CID spectra. Assignment of peptides into their source proteins was performed by X!tandem’s algorithm, and we chose to use its primary protein assignments only. Protein level intensities were the sum of the intensities of member peptides, expressed a log_2_ scale for simple differential analysis.

High confidence protein identifications were based on detection of at least two distinct peptides each with log_10_ expectation scores of −1.5 or lower, roughly equivalent to a protein-level log_10_ expectation score of −3.0 or lower. This yielded the identification of 1154 proteins at the start of CPB and 915 proteins at 1-h into CPB surgery. The total number of unique proteins observed was 1325, with 744 proteins identified in both samples (Additional file 2: Table S2). However, over a third of the population of the proteins identified (581) were seen exclusively at either the start of CPB or 1-h into CPB, rendering them ambiguous for quantitative analysis. This result highlights the challenge of comparative analysis of complex samples by mass spectrometry which arises as a result of the stochastic nature of instrument dependent data acquisition. Given the significant amount of time and effort committed to performing 2D DDA/IDA analysis and the variable complementarity of coverage between samples this was felt to be a suboptimal approach because such outcomes make it unclear if “missing” proteins in a sample were exclusively expressed in one set of the samples or if the peptides were simply not selected in one of the analyses. This motivated us to examine the feasibility of using a hyper reaction monitoring data independent approach [15].

As described in the Methods and Materials, we generated a large SWATH library by combining an existing human protein transition library [23] (henceforth called the “10K-library”) and the results of our larger-scale multiple 2D-LC–MS analysis of urines from cardiac bypass patients. Our approach was to replace our observed DDA/IDA transitions with their 10 K-library counterparts for the peptides common to both library modes (and across their multiple charge state versions), and retain transition patterns for peptides observed in our samples’ library but outside the 10K-library. The 10K-library’s unitless iRT retention scale [26] was linearly mapped into retention time using a simple regression, exhibiting an excellent correlation of R^2^ > 0.99. The combined library contained 18,628 peptides spanning 3620 proteins; of these 3429 peptides spanning 934 proteins were not part of the original 10K-library but were detected in our 2D analysis of urine samples (Additional file 3: Table S3).

### SWATH analysis

We applied the same two-peptide rule for SWATH quantitation as we did for the 2D DDA/IDA analysis, giving 4755 peptides spanning 730 proteins. This result suggested a remaining 1295 proteins were potentially quantifiable in SWATH, but by only a single peptide each. However, as quantitation based on a single peptide is less reliable these peptides were excluded from subsequent analysis. The complete peptide extraction report is available for additional exploration (see Availability of data and materials).

A total of 730 SWATH-quantifiable proteins were identified in the two sample pools, 552 of these also had quantitation values in both of the 2D LC runs. In addition there were 131 proteins that could be quantified using SWATH but not using the DDA/IDA derived data. These “gap filling” readouts indicated that SWATH presented the potential to differentially access approximately 22 % of the proteins that the 2D IDA dataset saw as incomplete (Additional file 3: Table S3).

### Combined DDA/IDA and SWATH quantitation approaches

The combined DDA/IDA and SWATH quantitation data were used to complement the quantitative analysis of each approach. The correlation between log_2_ SWATH and DDA/IDA protein expression values within a time point was moderate (R^2^ = 0.794 for start of CPB across 651 common proteins; R^2^ = 0.736 for 1-h into CPB across 584 common proteins), suggesting a general agreement between the readout technologies (Additional file 4: Table S4).

**Fig. 2:**
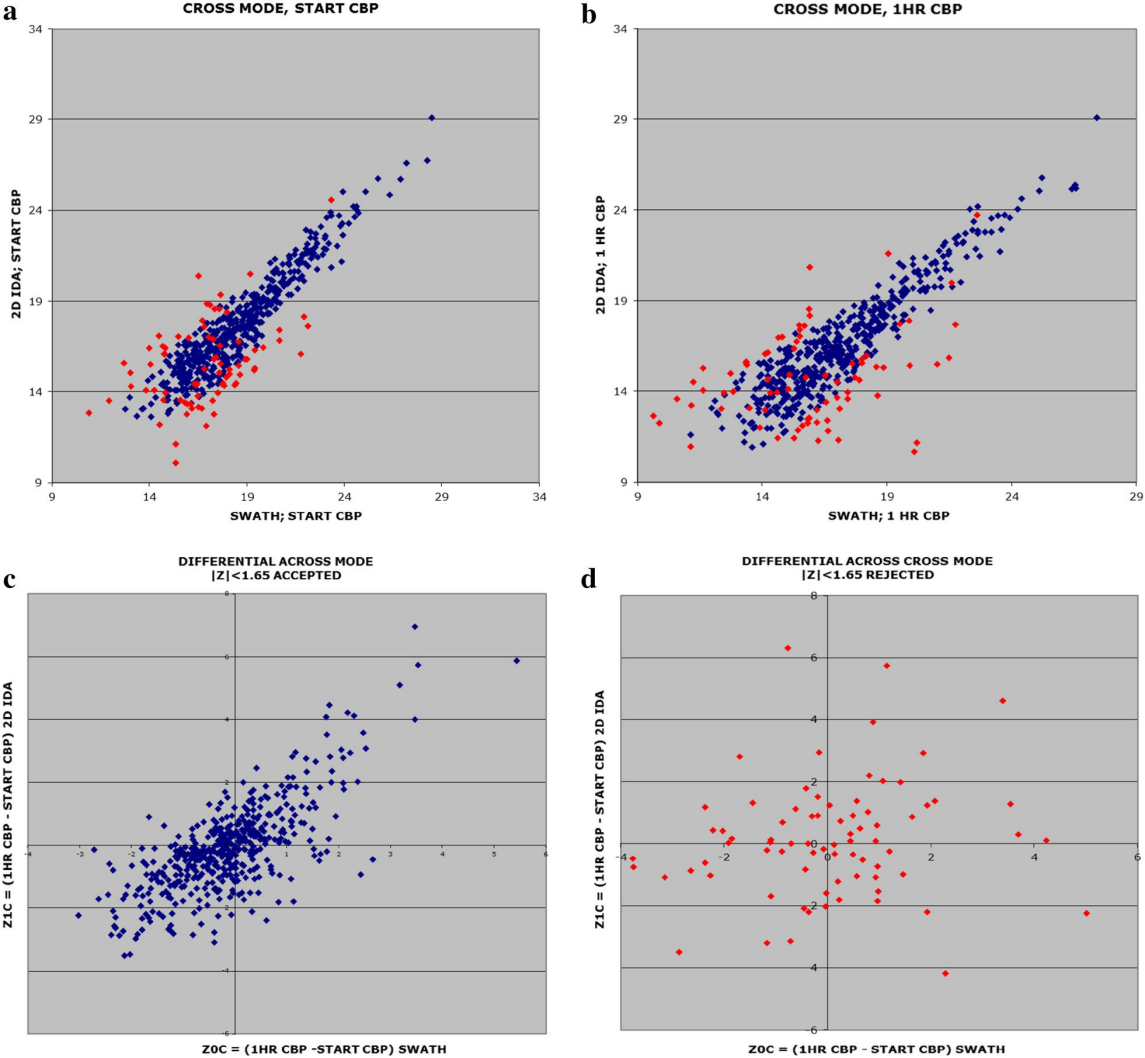
The use of protein level intensity correlations between 2D DDA/IDA and SWATH runs to improve quantitation reliability. Comparison of log_2_ protein intensities **a** START of CPB **b** 1-hour CPB. **c** The expression of differences between SWATH and DDA/IDA readouts (at the same time point) as normalized Z-scores identifies proteins that show discordance between the two methods. This provides a basis for discarding the proteins with |Z| > 1.65 in either population (as shown by the *red dots*), the resulting 475 common proteins (86 % of the 552 protein initial list; the *blue dots*). This improved the cross-technology difference correlation to R^2^ = 0.549. **d** The correlation of difference values for the 77 proteins rejected by this filtering step, shown is very low (R^2^ = 0.01)

Applying a population filtering to these “acquisition technology-replicates” improved our quantitation reliability with a modest loss of total quantified proteins. By expressing the difference between the SWATH and DDA/IDA readouts (at the same time point) as normalized Z-scores, then discarding proteins with |Z| > 1.65 in either population, the resulting 475 common proteins (86 % of the 552 protein initial list) improved the cross-technology difference correlation to R^2^ = 0.549, while the correlation of difference values for the 77 proteins rejected by this filtering step is low (R^2^ = 0.01) (Fig. 2).

These results were encouraging considering that SWATH runs consumed approximately one-sixth of the instrument acquisition time, with one-third amount less sample preparation time as their 2D-DDA/IDA counterparts and fewer steps to potentially compromise quantitation. Going forward, the SWATH ion library will be applied to broader AKI studies for the analysis of individual patient urine samples, where the subsequent “large-N” readout sets are amendable to more sophisticated statistical methods.

The supportive filtered population of 475 proteins formed the basis of our biological interpretation. In the absence of sampling statistics, we chose a fairly severe cut-off of threefold (log_2_ 1.59) protein expression difference required in both DDA/IDA and SWATH readouts. This gave 22 up-regulating proteins and 26 down-regulating proteins 1-h into CPB versus the start of CPB (Table 2).

**Table 2:** Correlation filtered 2D DDA/IDA and SWATH protein differences of magnitude greater than 1.59 (threefold up regulating at CPB-1-hour) from both methods marked in (A) and less than −1.59 (threefold down regulating at CPB-1-hour) marked in (B)

SWATH (log_2_ intensity ratio)	DDA/IDA (log_2_ intensity ratio)	UNIPROT entry	Description
(*A*)			
2.53	3.09	P01023	Alpha-2-macroglobulin
1.78	3.53	P04083	Annexin A1
2.05	3.04	P04003	C4b-binding protein alpha chain
3.53	5.73	P00915	Carbonic anhydrase 1
1.82	4.47	P49913	Cathelicidin antimicrobial peptide
1.87	2.36	P36222	Chitinase-3-like protein 1
3.47	4.00	P08123	Collagen alpha-2(I) chain
1.84	2.82	P09871	Complement C1 s subcomponent
1.86	2.01	P02671	Fibrinogen alpha chain
2.37	2.03	P02675	Fibrinogen beta chain
1.76	4.09	P30043	Flavin reductase (NADPH)
1.64	1.84	P00738	Haptoglobin
2.1	1.79	P01860	Ig gamma-3 chain C region
2.09	1.99	P13645	Keratin, type I cytoskeletal 10
2.3	4.12	P02533	Keratin, type I cytoskeletal 14
3.48	6.95	P08779	Keratin, type I cytoskeletal 16
5.44	5.87	Q04695	Keratin, type I cytoskeletal 17
2.17	4.22	P13647	Keratin, type II cytoskeletal 5
2.09	2.79	P02788	Lactotransferrin
3.18	5.10	P14780	Matrix metalloproteinase-9
2.22	2.94	P05164	Myeloperoxidase
2.48	3.59	P05109	Protein S100-A8
(*B*)			
−1.82	−1.94	P02763	Alpha-1-acid glycoprotein 1
−2.35	−2.10	P05090	Apolipoprotein D
−2.14	−3.50	P55287	Cadherin-11
−2.23	−2.87	P19022	Cadherin-2
−1.64	−1.80	P49747	Cartilage oligomeric matrix protein
−2.39	−2.86	P39059	Collagen alpha-1(XV) chain
−1.66	−2.32	P08174	Complement decay-accelerating factor
−1.64	−2.43	Q9HCU0	Endosialin
−1.92	−2.97	P17900	Ganglioside GM2 activator
−3.02	−2.24	Q96RW7	Hemicentin-1
−2.31	−2.30	O75144	ICOS ligand
−2.31	−2.63	Q9BRK3	Matrix-remodeling-associated protein 8
−1.72	−2.20	P08571	Monocyte differentiation antigen CD14
−1.78	−1.62	Q9BXP8	Pappalysin-2
−2.63	−1.72	P0DJD8	Pepsin A-3
−2.16	−1.72	Q6UXB8	Peptidase inhibitor 16
−1.7	−2.24	Q9HCN6	Platelet glycoprotein VI
−1.92	−1.60	P07602	Prosaposin
−1.88	−2.83	Q8WZ75	Roundabout homolog 4
−1.79	−2.70	O00241	Signal-regulatory protein beta-1
−2.02	−3.47	P19320	Vascular cell adhesion protein 1
−2.16	−2.73	Q6EMK4	Vasorin
−1.79	−2.34	Q12907	Vesicular integral-membrane protein VIP36
−2.33	−2.58	Q96DA0	Zymogen granule protein 16 homolog B

These formed the basis for our biological analysis

### Comparative analysis of the urinary proteome in patients undergoing CPB

The proteomic changes observed in these cardiac surgery patients were felt to reflect physiological responses to the trauma of the surgery and the associated bypass process, including ischemia reperfusion injury.

The majority of proteins that displayed altered expression after 1-h of CPB were localised to exosomes (Table 3). These findings are consistent with the observation that exosomes are derived from multivesicular bodies which fuse with renal tubular epithelial cell membranes before being secreted into the urine [27]. Urinary exosomes are typically isolated by differential centrifugation prior to proteomic analysis, and identification of exosomal proteins here suggests that these methods may identify low abundance proteins; although proteome overlap between whole urine and exosomes has been reported [28]. These results could suggest that the repertoire of exosomal proteins changed during CPB but the process of exosome generation continued.

**Table 3:** GO cellular components enriched in differentially regulated proteins

Term	Up regulated		Down regulated	
	Genes	*p* value Bonferroni	Genes	*p* value Bonferroni
Extracellular exosome	20	4.41 × 10^−14^	23	4.99 × 10^−18^
Extracellular membrane-bounded organelle	20	4.41 × 10^−14^	23	4.99 × 10^−18^
Extracellular organelle	20	4.79 × 10^−14^	23	5.50 × 10^−18^
Extracellular region part	21	9.89 × 10^−14^	22	3.22 × 10^−13^
Membrane-bounded vesicle	20	2.81 × 10^−12^	23	6.18 × 10^−16^
Extracellular region	21	3.59 × 10^−12^	22	1.33 × 10^−11^
Vesicle	20	5.11 × 10^−12^	23	1.24 × 10^−15^
Extracellular space	15	1.82 × 10^−11^	11	2.95 × 10^−5^
Blood microparticle	6	1.40 × 10^−6^	*	*
Cytoplasmic membrane-bounded vesicle lumen	5	3.08 × 10^−5^	*	*
Vesicle lumen	5	3.27E × 10^−5^	*	*

Only cellular components enriched with a *p* value of 10^−5^ or less are reported. Analysis was performed using String database [37]

The up-regulated proteins were enriched for humoral immune responses (Table 4), which included predominantly innate features as outlined by the list of proteins involved (e.g. complement, lactotransferrin, myeloperoxisae and cathelicidin anti-microbial peptide). It is noteworthy that Chitinase-3-like protein 1 (CHI3L1, also called YKL-40) was also increased at 1-h post initiation of CPB. CHI3L1 is a member of the chilectin subfamily of the 18 glycosyl hydrolase family. This protein has retained the ability to bind heparin, collagen and chitin but it is enzymatically inactive [29]. It has been shown to have a number of immunostimulatory properties for the adaptive immune system as well as pro-inflammatory activities [29]. Increased levels of CHI3L1 have been associated with loss of renal function in diabetes, and renal or cardiac transplantation patients [30–33]. Our present observations suggest that CHI3L1 levels also increase in patients undergoing cardiac bypass surgery potentially as a result of intra operative subclinical ischemia–reperfusion injury.

**Table 4:** GO processes enriched in upregulated proteins

GO term	Humoral immune response	Immune system process	Antibacterial humoral response	Protein activation cascade	Antimicrobial humoral response	Defense response to other organism	Response to inorganic substance	Defense response to bacterium
Genes	6	12	4	5	4	7	8	6
*p* value Bonferroni	1.87 × 10^−5^	5.08 × 10^−5^	1.87 × 10^−5^	1.87 × 10^−5^	1.98 × 10^−5^	5.08 × 10^−5^	1.87 × 10^−5^	5.08 × 10^−5^
ANXA1		X					X	
A2 M				X				
C1S	X	X		X				
C4BPA	X	X		X				
CAMP	X	X	X		X	X		X
COL1A2		X						
FGA	X	X	X	X	X	X	X	X
FGB	X	X	X	X	X	X	X	X
HP		X				X	X	X
LTF	X	X	X		X	X		X
KRT10							X	
KRT14							X	
KRT16		X						
MPO		X				X	X	
S100A8		X				X	X	X

Note only those terms with Bonferroni corrected *p* values of 10^−5^ or lower are listed. The proteins assigned to the process are indicated. Analysis was performed using String database [37]

These up-regulated proteins also contained a number of extracellular components, although these proteins appeared to derive more from vesicle or granule contents. ECM reorganization was a process characterized by the up-regulated proteins, including elevation of urinary MMP9. These findings are consistent with reports that demonstrate elevated urinary MMP7 and renal MMP2 expression in ischemia reperfusion injury of renal allografts [4, 34]. While there was one diabetic patient in the cohort, it is unlikely that the acute intraoperative elevation of urinary MMP9 observed over 1-h of CPB was related to undocumented diabetic nephropathy [35].

Significantly, the up-regulated proteins also reflected general activation/regulation of innate immunity (C4b, lactotransferrin, protein S100-A8, myeloperoxidase) which is consistent with rapid, generic responses to cardiac surgery-induced tissue injury. Interestingly, there were also changes suggesting that the adaptive immune response might be down-regulated following 1-h on CPB, a process not previously described. Taken together, these findings corroborate pre-clinical models of the immune responses to renal ischemia reperfusion injury, but further expand on them to demonstrate that repair and re-modeling processes may be initiated sooner than previously thought [36].

It should be emphasised that the criteria used for the selection of proteins with significant changes between the two time points was based on the requirement that the protein was identified in both SWATH and DDA/IDA analyses of the same sample, and the changes had to be of comparable magnitudes. These represent very stringent criteria to provide confidence regarding the presence and magnitude of the change using two independent analytical methods. This was not meant to imply that other quantitative changes predicted by observations made in only one of the approaches (DDA/IDA or SWATH) may not also be valid. However, the confidence of such changes is lower and would require validation using alternate methodologies.

## Conclusions

Our results provide a method for analyzing the urinary proteomic changes in adult patients undergoing cardiac surgery. Unlike the original FASP method the current procedure does not use SDS. The method requires limited sample processing to remove interfering substances and generates mass spectrometry ready samples. The design is such that it can be readily applied to large numbers of clinical samples with the potential for automation. The introduction of SWATH into the workflow offers several benefits to the analysis. The most significant aspect is the ability to consistently report on the same panel of >700 proteins in each analysis. This allows for more confident comparisons between samples, while keeping both the sample quantity requirements and the mass spectrometer instrument time relatively low. Although SWATH does not currently offer the analytical depth provided by 2D-LC–MS/MS analyses, it may be possible to extend the level of quantitation through longer chromatographic run times or via pre-fractionation steps prior to SWATH-MS. There have been several comparisons to suggest that SWATH provides comparable quantitative analysis to other methods [22]. The approach outlined here is particularly relevant to the analysis of sequential urines from patients in various clinical conditions, including intraoperative samples.

## Additional files

10.1186/s12014-016-9118-9 The use of custom retention alignment peptides to map peptide retention times from 2D DDA/IDA runs into the SWATH extraction library, and their corresponding SWATH extracted retention times.
10.1186/s12014-016-9118-9 The log(2) protein values for DDA/IDA at time-points START and CPB-1-hour.
10.1186/s12014-016-9118-9 (A) An overview of the peptide and protein identifications and resulting differential quantitation for the 2D-LC DDA/IDA and SWATH run datasets. (B) The impact of the application of a DDA/IDA and SWATH intensity correlation filtering to the differential analysis.
10.1186/s12014-016-9118-9 Correlation filtered 2D DDA/IDA and SWATH protein difference values.

## Abbreviations

CPB: cardiopulmonary bypass
MS: mass-spectrometry
FASP: filter assisted sample preparation
SWATH: Sequential Window acquisition of All Theoretical fragment ion spectra
eGFR: estimated glomerular filtration rate
MWCO: molecular weight cut-off ultra-centrifugal filters
HPLC: high-performance liquid chromatography
2D-LC–MS/MS: two dimensional liquid chromatography mass-spectrometry
